# RNA–Polymer Conjugates via Direct Incorporation of the Chain Transfer Agent and PET–RAFT Polymerization

**DOI:** 10.1021/acs.biomac.5c00838

**Published:** 2025-08-25

**Authors:** Xiaolei Hu, Jaepil Jeong, Hironobu Murata, Rongguan Yin, Subha R. Das, Krzysztof Matyjaszewski

**Affiliations:** Department of Chemistry, 6612 Carnegie Mellon University, Pittsburgh, Pennsylvania 15213, United States

## Abstract

Covalent conjugation of RNA with synthetic polymers has emerged as a powerful approach for creating bioconjugates with synergistically enhanced properties. However, conventional methods require solid-phase synthesis to preinstall functional groups in RNA, significantly limiting practical applications. Here, we present a novel approach for synthesizing RNA–polymer conjugates via direct incorporation of chain transfer agent (CTA) into RNA through acylation chemistry and reversible addition–fragmentation chain transfer (RAFT) polymerization. A CTA-functionalized acyl imidazole reagent was synthesized to facilitate direct and covalent modification of various RNAs by reacting with their 2′-hydroxyl groups. Subsequent RAFT polymerization using RNA–CTA as a macro-CTA enabled direct grafting-from RNA, yielding RNA conjugates with controlled molecular weight and low dispersity. Notably, this postsynthetic modification strategy was successfully extended to modify biomass RNA, yielding thermoresponsive conjugates and biodegradable hydrogels. Overall, this advance allowed for the direct modification of synthetic and biomass RNAs, significantly enhancing the accessibility of functional RNA–polymer materials.

## Graphical abstract

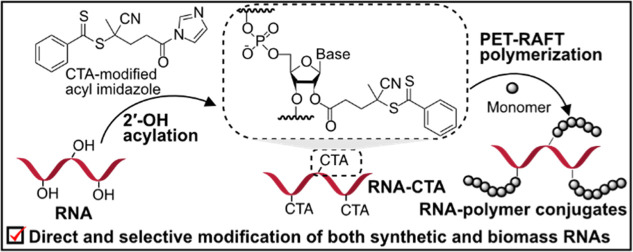

## Introduction

Covalent conjugation of nucleic acid (NA) with synthetic polymers has emerged as a powerful approach for creating novel bioconjugates.^1–3^ These hybrid materials leverage the biological activities and programmable base pairing of NA (e.g., RNA) and tunable and diverse properties of synthetic polymers, enabling wide-ranging applications in biomedicines and nanomaterials.^4–9^ Current synthetic approaches for polymer–NA conjugates can be divided into two categories: “grafting-to” and “grafting-from” strategies. The “grafting-to” approach directly conjugates presynthesized polymers with NA through various coupling reactions, such as azide–alkyne cycloaddition,^8,10^ amidation,^11^ Michael addition,^12^ and thiol/disulfide exchange.^13^ While this straightforward method fabricates bioconjugates with a predetermined structure and molecular weight (MW), it suffers from several limitations: low coupling efficiency and yield due to steric hindrance between macromolecular reactants, challenges in purifying bioconjugates from unreacted precursors, and incompatible solubility between hydrophilic NA and hydrophobic polymer.^14^

The “grafting-from” approach has recently emerged as a promising alternative, thanks to its high yield and facile purification of resulting bioconjugates from unreacted monomers.^15^ This strategy typically employs reversible-deactivation radical polymerization (RDRP) techniques,^16^ particularly atom transfer radical polymerization (ATRP) and reversible addition–fragmentation chain transfer (RAFT) polymerization.^17–23^ These powerful RDRP methods enable controlled polymer growth from NA modified with radical regulating groups, typically an alkyl halide initiator for ATRP and chain transfer agent (CTA) for RAFT, providing precise control over the MW, architecture, and composition of conjugated polymers. Particularly, this “grafting-from” process is significantly facilitated by recent advances in oxygen-tolerant photoinduced RDRP, overcoming the quenching of propagating radicals by O_2_ during the RDRP process that otherwise would require an inert atmosphere using a complicated and expensive setup.^24–26^ This development allows polymerization to proceed even at ambient conditions and microliter scales, making it more accessible to nonexperts and broader and practical applications in this emerging field. However, the “grafting-from” approach faces another limitation as it requires preinstallation of functional groups (e.g., amine, thiol, azide, and alkyne) in NA through solid-phase synthesis before coupling with CTA or initiator. Additionally, this solid-phase modification restricts the approach to synthetic RNA substrates, excluding other functional RNAs (e.g., mRNAs). Moreover, the restriction to expensive and less-available synthetic RNA significantly limits the fabrication of functional biomaterials for diverse applications. The key to addressing these challenges is developing a robust strategy for the direct incorporation of RDRP-mediating reagents.

To overcome these challenges and expand RNA scope for conjugates, we were inspired by the acylation chemistry for RNA modification involving its intrinsic 2′-hydroxyl (2′-OH) group.^27^ This postsynthetic reaction has been widely used as one of the most effective approaches for RNA modification owing to its high reactivity, selectivity, biocompatibility, and simplicity. It has even been exploited to directly regulate biological activities in vitro and in vivo.^28^ We envision that this robust acylation chemistry would allow the development of a direct and universal approach for incorporating RDRP mediating reagents into RNAs and, thereafter, the synthesis of RNA–polymer conjugates. Our group recently applied it for postsynthetic RNA modification with alkyl halide ATRP initiators and subsequent controlled polymer growth via photoinduced ATRP.^29^ Another important RDRP technique, RAFT polymerization, utilizes organic thiocarbonate CTA for mediating radical propagation via a degenerative transfer mechanism and offers a promising solution to residual metallic catalyst contamination. Furthermore, polymerization-induced self-assembly via the RAFT process allows one-pot fabrication of functional NA–polymer nanoparticles for gene delivery and regulation,^19,30^ overcoming long-standing challenges in synthesizing amphiphilic biconjugates.^31^ However, current RAFT-based approaches still rely on multistep modification reactions with solid-phase synthesis and are applicable only to synthetic RNA due to limited synthetic approaches as well as the instability of thiocarbonate-based CTA (Scheme 1A).

**Scheme 1:**
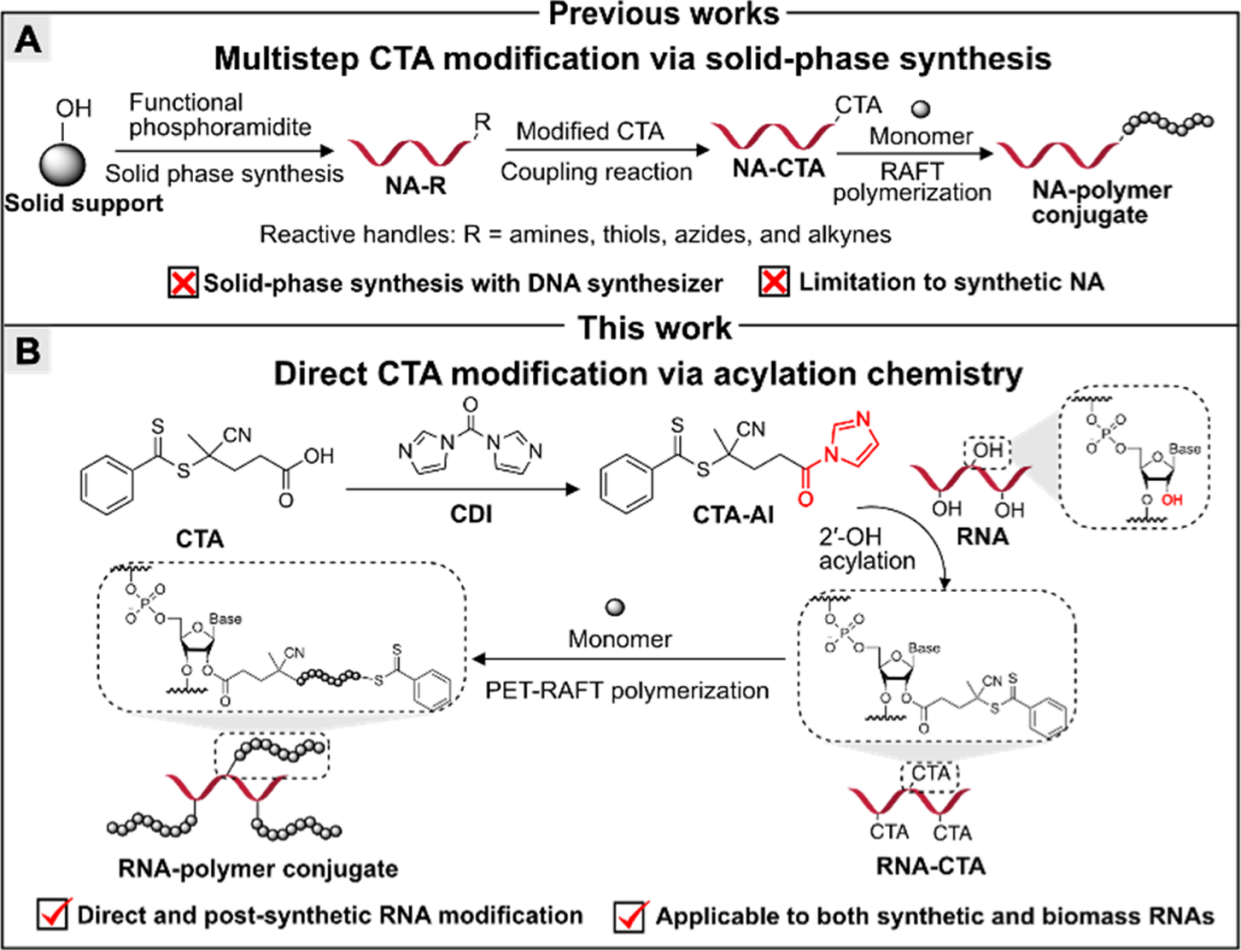
(A) Summary of a Previous Work Involving Multistep CTA Modification via Solid-Phase Modification for “Grafting-from” NA Using RAFT Polymerization; (B) This Work: Direct RNA Modification via a CTA-Functionalized Acyl Imidazole (CTA-AI) for “Grafting-from” RNA via PET-RAFT Polymerization

Motivated by these, herein, we present a universal and facile approach for synthesizing RNA–polymer conjugates and hydrogels by combining direct and covalent modification of RNA with CTA and subsequent controlled growth of polymer via RAFT polymerization. Our strategy starts with the design and synthesis of CTA-modified acyl imidazole reagent (CTA-AI) using commercially available reagents 4-cyano-4-(phenylcarbonothioylthio)­pentanoic acid (CPADB) and 1,1′-carbonyldiimidazole (CDI, Scheme 1B). Nucleophilic substitution of CTA-AI by the reactive 2′-OH of RNA enables direct covalent conjugation of CTA within synthetic RNAs with varying sequences. Among RAFT polymerizations initiated by various external stimuli, the photoinduced electron/energy transfer process was selected for facilitating the direct growth of polymer from RNA due to its oxygen tolerance, small-scale reaction, and suppressed formation of free polymer chains,^32,33^ affording well-defined RNA–polymer conjugates. Moreover, this postsynthetic approach allows the grafting of diverse polymers from widely available biomass RNA, enabling the fabrication of stimuli-responsive conjugate nanoparticles and biodegradable hydrogels with potential biomedical applications.^9,34^

## Experimental Section

### Materials

Unless otherwise noted, all chemicals were purchased from commercial sources and used as received. 4-Cyano-4-(phenylcarbonothioylthio)­pentanoic acid (CPADB), carbonyldiimidazole (CDI), 6-acrylamidohexanoic acid, biomass RNA extracted from torula yeast (type VI), oligo­(ethylene glycol) methyl ether methacrylate (average *M*_n_ = 500, OEOMA_500_), poly­(ethylene glycol) dimethacrylate (average *M*_n_ = 750, PEGMA_750_), eosin Y (EY), triethanolamine (TEOA), 3-hydroxypicolinic acid (HPA), GelRed (10,000× in DMSO), and 96-well plates were purchased from Sigma-Aldrich. All monomers were passed through a column of basic alumina to remove the inhibitor prior to use. 10× Phosphate-buffered saline (10× PBS) was purchased from Thermo Fisher Scientific. Water (HPLC grade), dimethylformamide (DMF, HPLC grade), tetrahydrofuran (THF, HPLC grade), dimethyl sulfoxide (DMSO, HPLC grade, anhydrous), and isopropyl alcohol were purchased from Fisher Chemical. D_2_O and DMSO-*d*_6_ were purchased from Cambridge Isotope Laboratories, Inc. Ribonucleotide (DNA) phosphoramidites and the CPG solid support for RNA synthesis were purchased from Chemgenes and Glen Research. SYBR Gold dye (10,000× in DMSO) was purchased from Invitrogen. Sep-Pak C18 cartridge was purchased from Waters.

### Synthesis of CTA-AI Acylation Reagent

CPADB (0.73 g, 4.5 mmol) and CDI (1.26 g, 4.5 mmol) were dissolved in DMSO (anhydrous) separately and mixed. The final volume of the reaction mixture was brought up to 3 mL and incubated under gentle shaking for 30 min to obtain the CTA-functionalized acyl imidazole reagent (CTA-AI) stock solution (1.5 M).

### General Procedure for Incorporation of CTA in Oligonucleotide RNA

Stock solutions of the oligo RNA_21_ substrate in nuclease-free H_2_O (6.6 mM, 30 μL), CTA-AI in DMSO (1.5 M, 200 μL), and nuclease-free H_2_O (770 μL) were mixed thoroughly in an Eppendorf tube. The final concentrations were RNA substrate (0.2 mM), CTA-AI (300 mM), and DMSO (20% v/v). The mixture was incubated overnight at room temperature under gentle shaking. The reaction mixture was subjected for centrifugation (13,000 *g*, 10 min) at 4 °C. The modified RNA in the resulting supernatant was then precipitated by adding 0.1× volume of sodium acetate (2 M) and 1.5× volume of isopropanol at −20 °C overnight. The precipitates were collected after centrifugation (13,000 *g*, 30 min) at 4 °C and redispersed in 200 μL of nuclease-free water. The precipitation purification process was repeated 3 times to ensure complete removal of unconjugated CTA derivatives. The concentration of obtained RNA_21_–CTA was determined by measuring absorbance at 260 nm (*A*_260_) based on the appropriate extinction coefficient calculated by the Oligo Analyzer (Integrated DNA Technologies). MALDI-TOF spectra of the modified RNA were recorded using 3-hydroxypicolinic acid (HPA) as the matrix, which was prepared in 50% acetonitrile/water containing 10 mg/mL of diammonium hydrogen citrate. PAGE analysis was carried out using a 12% native polyacrylamide gel at 120 V for 60 min. SYBR Gold was used for visualization.

### General Procedure for Incorporation of CTA in Biomass RNA

Biomass RNA extracted from torula yeast (40 mg), CTA-AI in DMSO (1.5 M, 200 μL), and nuclease-free H_2_O (800 μL) were mixed and placed in an Eppendorf tube and were then incubated overnight at room temperature under gentle shaking. Next, the reaction mixture was precipitated by using 0.1× volume of sodium acetate (2 M) and 1.5× volume of isopropanol at −20 °C overnight. The precipitates were collected after centrifugation (13,000*g*, 30 min) at 4 °C and were redispersed in 1 mL of nuclease-free water. The precipitation purification process was repeated 3 times to ensure complete removal of unconjugated CTA derivatives. The concentration of the bmRNA–CTA solution was determined by measuring *A*_260_ (extinction coefficient = 40 (μg/mL)^−1^ cm^–1^).

### General Procedure for EY-Catalyzed PET–RAFT Polymerization of OEOMA_500_

First, stock solutions of OEOMA_500_ (600 mM in H_2_O), EY (1.5 mM in H_2_O), TEOA (10 mM in H_2_O), and CPADB (50 mM in DMSO) were prepared. A typical polymerization “cocktail” solution (250 μL) is then prepared as follows: OEOMA_500_ stock (125 μL), EY stock (2.5 μL), TEOA stock (22.5 μL), CPADB (2.5 μL), DMSO (3.75 μL), H_2_O (68.75 μL), and 10× PBS solution (25 μL) were thoroughly mixed. The final concentrations were OEOMA_500_ (300 mM), EY (15 μM), TEOA (0.9 mM), CPADB (0.5 mM), DMSO (1.5% v/v), and 1× PBS. The solution was transferred to glass inserts (250 μL) and irradiated under green light (520 nm, 3.7 mW/cm^2^) for 30 min. The resulting sample was withdrawn and analyzed by the ^1^H NMR and SEC-MALS techniques.

### EY-Catalyzed PET-RAFT for Synthesizing the RNA_21_–Polymer Conjugate

First, stock solutions of OEOMA_500_ (600 mM in H_2_O), EY (1.5 mM in H_2_O), and TEOA (10 mM in H_2_O) were prepared. A typical polymerization “cocktail” solution (100 μL) is then prepared as follows. OEOMA_500_ stock (50 μL), EY stock (1 μL), TEOA stock (9 μL), RNA_21_–CTA (3.09 mM, 1.62 μL), H_2_O (28.4 μL), and 10× PBS solution (10 μL) were then mixed. The final concentrations were OEOMA_500_ (300 mM), EY (15 μM), TEOA (0.9 mM), RNA_21_–CTA (50 μM), and 1× PBS. The solution was transferred to the glass inserts and irradiated under green light (520 nm, 3.7 mW/cm^2^) for 30 min. The resulting sample was withdrawn and analyzed by ^1^H NMR and SEC-MALS techniques.

### EY-Catalyzed PET-RAFT for Synthesizing the bmRNA–Polymer Conjugate

First, stock solutions of OEOMA_500_ (600 mM in H_2_O), EY (1.5 mM in H_2_O), and TEOA (100 mM in H_2_O) were prepared. A typical polymerization “cocktail” solution (250 μL) is then prepared as follows: OEOMA_500_ stock (125 μL), EY stock (2.5 μL), TEOA stock (2.25 μL), bmRNA–CTA (112.8 mg/mL, 1.1 μL), DMSO (3.75 μL), H_2_O (90.39 μL), and 10× PBS solution (25 μL) were then mixed. The final concentrations were OEOMA_500_ (300 mM), EY (15 μM), TEOA (0.9 mM), bmRNA–CTA (0.5 mg/mL), DMSO (1.5% v/v), and 1× PBS. The solution was transferred to the glass inserts and irradiated under green light (520 nm, 3.7 mW/cm^2^). At different time intervals (10, 20, 40, 60, and 80 min), the samples were withdrawn and analyzed by ^1^H NMR and SEC-MALS techniques.

### Chain Extension from bmRNA-*b*-pOEOMA_500_

For the synthesis of bmRNA-*b*-pOEOMA_500_-*b*-pOEOMA_500_, polymerization solution (250 μL) was prepared based on the general procedure for EY-catalyzed PET-RAFT with final concentrations of OEOMA_500_ (300 mM), EY (15 μM), TEOA (0.9 mM), biomass RNA–CTA (6 mg/mL), DMSO (1.5% v/v), and 1× PBS. The solution was transferred to a glass inserts and irradiated under green light (λ = 520 nm, 3.7 mW/cm^2^) at room temperature for 60 min. After polymerization, the sample was withdrawn and analyzed by ^1^H NMR and SEC-MALS techniques. Next, the crude macroinitiator sample taken from the postpolymerization solution (20 μL) was directly mixed with the OEOMA_500_ stock (125 μL), EY stock (2.5 μL), TEOA stock (2.25 μL), DMSO (3.75 μL), H_2_O (90.39 μL), and 10× PBS solution (25 μL). The solution was transferred to the glass inserts and irradiated under green light (520 nm, 3.7 mW/cm^2^) for 60 min. Finally, the resulting sample was withdrawn and analyzed by the ^1^H NMR and SEC-MALS techniques.

### Synthesis of bmRNA-*b*-pNIPAm

First, stock solutions of NIPAm (600 mM in H_2_O), EY (1.5 mM in H_2_O), and TEOA (100 mM in H_2_O) were prepared. A typical polymerization “cocktail” solution (250 μL) is then prepared as follows: NIPAm stock (125 μL), EY stock (2.5 μL), TEOA stock (2.25 μL), bmRNA–CTA (112.8 mg/mL, 1.1 μL), DMSO (3.75 μL), H_2_O (90.39 μL), and 10× PBS solution (25 μL) were then mixed. The final concentrations were NIPAm (300 mM), EY (15 μM), TEOA (0.9 mM), bmRNA–CTA (0.5 mg/mL), DMSO (1.5% v/v), and 1× PBS. The solution was transferred to the glass inserts and irradiated under green light (520 nm, 3.7 mW/cm^2^) for 60 min. The sample was withdrawn and analyzed by ^1^H NMR and SEC-MALS techniques. The rest of the sample was purified by precipitation at 50 °C and centrifugation (13,000*g*, 5 min). The purified conjugate was stained SYBR gold (λ_ex_: 495 nm, λ_em_: 537 nm) and analyzed by a microplate reader.

### Synthesis of the bmRNA-Derived Acrylamide Cross-Linker (bmRNA-Am)

The procedure was adapted based on our previous report.^35^ First, 6-acrylamidohexanoic acid (3.24 g, 20 mmol) and CDI (4.60 g, 20 mmol) were dissolved separately in anhydrous DMSO and mixed. The final volume of the reaction mixture was brought up to 20 mL and incubated under stirring for 30 min to obtain acrylamide-functionalized acyl imidazole reagent (Am-AI) stock solution (1 M). Next, biomass RNA extracted from torula yeast (40 mg), Am-AI (1 M, 400 μL), nuclease-free H_2_O (133 μL) were mixed welled in an Eppendorf tube and then incubated overnight at room temperature under gentle shaking. Next, the reaction mixture was precipitated by using 0.1× volume of sodium acetate (3 M) and 1.5× volume of isopropanol at −20 °C for overnight. The precipitates were collected after centrifugation (13,000*g*, 30 min) at 4 °C and were redispersed in 1 mL of nuclease-free water. The precipitation purification process was repeated 3 times. The concentration of purified bmRNA-Am solution was determined by measuring *A*_260_.

### Fabrication of the RNA–Polymer Hydrogel by EY-Catalyzed PET-RAFT and Enzymatic Degradation in FBS

As a typical example, a polymerization solution (100 μL) was prepared based on the general procedure for EY-catalyzed PET-RAFT with final concentrations of OEOMA_500_ (50 mM), bmRNA-Am (100 mg/mL), EY (15 μM), TEOA (0.9 mM), and bmRNA–CTA (1 mg/mL). The solution was first transferred to a 96-well plate mounted on a 96-point green light LED array (527 nm, 20 mW cm^–2^) at room temperature for 120 min. After polymerization, the resulting hydrogel was stained by incubation in GelRed solution (100×, 1.5 mL) overnight. Subsequently, the hydrogel was washed with water 3 times and incubated in fresh water overnight. Finally, the stained hydrogel was soaked in fetal bovine serum (FBS) (15%) in an aqueous buffer (3 mM MgCl_2_, 50 mM Tris–HCl, 75 mM KCl, and 10 mM DTT) at 37 °C to test the enzymatic degradation for 40 days. In the control group, the stained hydrogel was incubated in H_2_O for 40 days.

## Results and Discussion

### Selective and Postsynthetic Modification of RNA with CTA

Among different types of acylation reagents, acyl imidazole derivatives were selected due to their high reactivity and stoichiometric efficiency in RNA acylation.^36^ Based on this, the commercially available CPADB was selected as a model CTA because of its wide use in O_2_-tolerant PET-RAFT polymerization and compatibility with diverse photocatalysts. Additionally, its terminal carboxylic acid group can be readily activated by treatment with CDI in anhydrous DMSO to form a CTA-modified acyl imidazole (CTA-AI) for RNA modification (Scheme 1B). ^1^H NMR analysis confirmed the successful synthesis of CTA-AI (Figure S1).

To evaluate the efficacy of RNA modification via acylation chemistry, a 21-mer RNA (RNA_21_; see the sequence in Table S1) was treated with CTA-AI ([2′-OH]/[CTA-AI] = 30) in a DMSO/H_2_O mixture (20%, v/v) under gentle shaking overnight (Figure 1A). The reaction products were purified by three cycles of repeated precipitation from isopropanol to ensure complete removal of unreacted CTA-AI and byproducts. After ultracentrifugation, the modified RNA pellet was collected and redispersed in water, exhibiting a yellow color, which was in stark contrast to the colorless pristine RNA in both the solid and solution states (Figure S2A–D). This distinct color of modified RNA was similar to the yellow color of the CTA-AI solution (Figure S2E), suggesting the incorporation of CTA via acylation chemistry. To further confirm and quantify covalent modification, matrix-assisted laser desorption/ionization time-of-flight (MALDI-TOF) spectra of RNA were recorded before and after CTA-AI treatment (Figure 1B,C). The mass spectrum of modified RNA_21_ revealed the complete disappearance of the pristine RNA_21_ peak at 6653.4, accompanied by the appearance of multiple new peaks ranging from 6754.3 to 7512.6. Gaussian fitting of this distribution yielded a mean of 7289.5 with a standard deviation of 271.7. Unexpectedly, the MW of these new peaks (*M*_n,MALDI_) was lower than the theoretical values (*M*_n,th_) of RNA_21_–CTA adducts with a varying number of attached CTA (abbreviated as RNA_21_–CTA_*x*_, where *x* denotes the number of attached CTA per RNA, Table S2). It has been reported that the high-energy UV laser used in MALDI-TOF analysis caused fragmentation of the chain-end CTA group in polymers and DNA–CTA through cleavage of the labile C–S bond, resulting in a deviation from the theoretical MW.^20^ Indeed, such a UV-induced radical formation is commonly utilized for initiating RAFT polymerization, known as photoiniferter RAFT.^37^ Therefore, we hypothesize that the observed MW deviation could be attributed to the loss of the dithiobenzoic moiety of RNA–CTA_*x*_ following C–S bonds cleavage during the MALDI process,^20^ generating RNA_21_–CN_*x*_ adducts (Figure 1A). As shown in Table S2, the *M*_n,th_ of RNA_21_–CN_*x*_ correlate well with the new peaks observed in the MALDI-TOF spectrum after modification (Figure 1C), confirming the synthesized RNA_21_–CTA_*x*_ via acylation and its fragmentation during MALDI. The number of incorporated CTA per RNA_21_ was determined to range from 1 to 8, with an average of six CTAs, which is comparable to the modification degree of other reported acyl imidazole reagents under similar conditions.^29^ Note that the heterogeneous degree of modification and accompanying fragmentation resulted in side peaks and a relatively lower signal-to-noise ratio in the MALDI spectra of RNA–CTA adducts.

**Figure 1:**
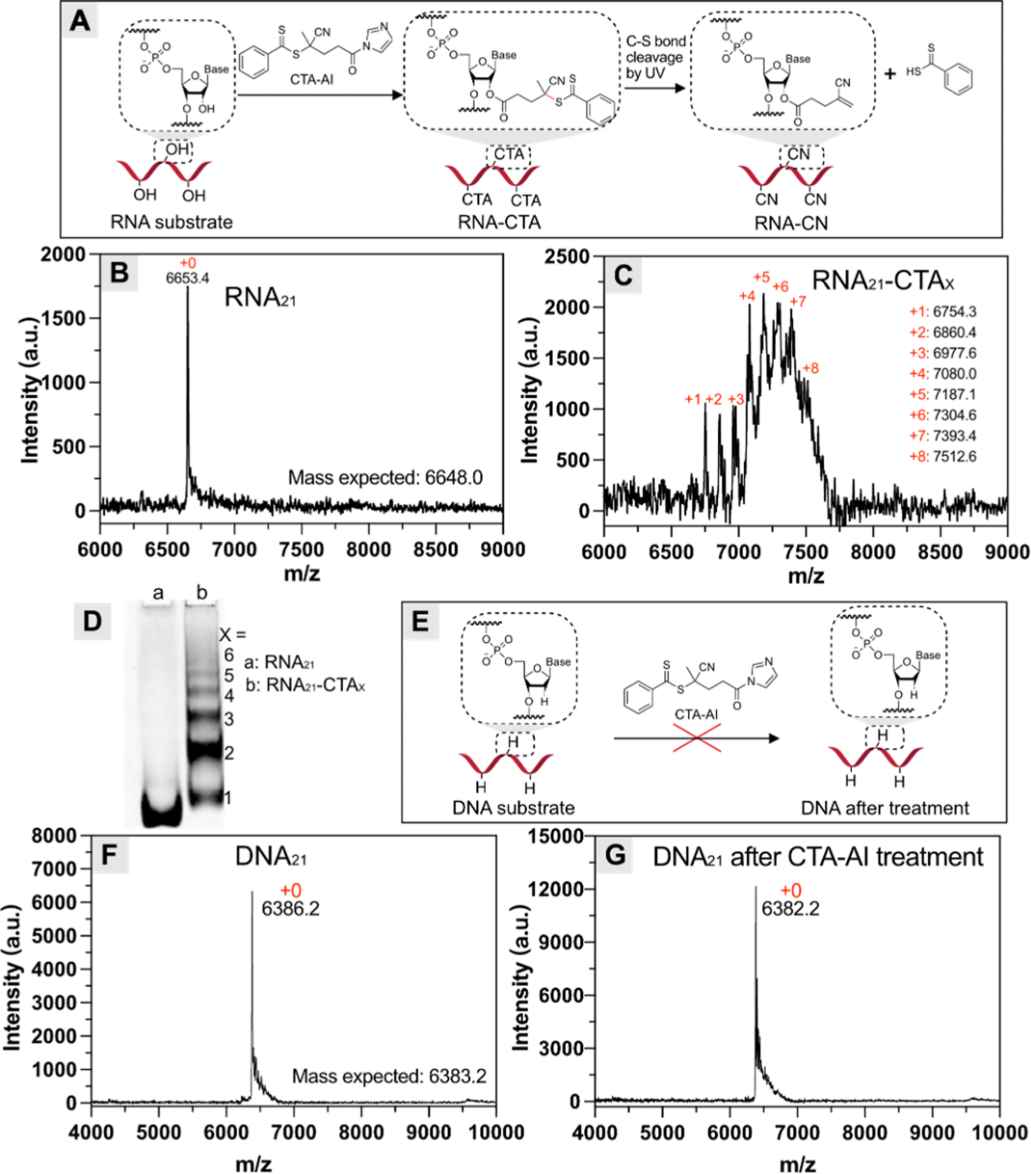
Selective RNA modification using CTA-AI. (A) Schematic illustration of modification of RNA with CTA and subsequent cleavage of the resulting RNA–CTA adducts during MALDI-TOF characterization. (B,C) MALDI-TOF spectra of RNA_21_ (B) before and (C) after treatment with CTA-AI. (D) PAGE analysis of RNA_21_ before and after CTA-AI treatment. (E) Schematic illustration of the treatment of DNA with CTA-AI. (F,G) MALDI-TOF spectra of DNA_21_ (F) before and (G) after treatment with CTA-AI.

Next, RNA acylation with CTA was further investigated by polyacrylamide gel electrophoresis (PAGE) analysis. As shown in Figure 1D, the original band corresponding to native RNA_21_ disappeared after treatment with CTA-AI, accompanied by the appearance of multiple new bands. This was attributed to the different mobility of the formed RNA_21_–CTA_*x*_ adducts with varying numbers of incorporated CTA per RNA,^28^ aligning with previous MALDI-TOF results. Additionally, compared to the original RNA_21_ with a characteristic peak at 260 nm, the UV–vis of RNA_21_–CTA_*x*_ showed a pronounced shoulder peak at 320 nm, originating from the attached CTA (Figure S3).^20^

Since the covalent modification of RNA with CTA via acylation reaction relied on its intrinsic 2′-OH group, we hypothesized that this approach would show high selectivity toward RNA over DNA. To test this hypothesis, DNA_21_ with a similar sequence as RNA_21_ (except for thymine replacing uracil, Table S1) was treated with CTA-AI under identical conditions (Figure 1E). In contrast to previous RNA modification, MALDI-TOF analysis of the treated DNA_21_ revealed the retention of the original DNA peak without any new peaks (Figure 1F,G), confirming the absence of CTA incorporation due to the lack of 2′-OH group in DNA.^28^

To examine the universality of our postsynthetic modification approach, we expanded our testing on two additional synthetic RNA substrates with different sequences, RNA_20_ and RNA_22_ (see the sequence in Table S1). MALDI-TOF analysis (Figures S4 and S5) confirmed successful CTA incorporation, with an average of four and six CTA moieties attached to RNA_20_ and RNA_22_, respectively. Additionally, the consistent MW deviation between *M*_n,MALDI_ and *M*_n,th_ observed in these cases during MALDI-TOF further validated the universal fragmentation due to the intrinsic labile C–S bond in the attached CTA group. These results demonstrated that our acylation approach enables the selective and facile incorporation of CTA into RNA through a postsynthetic manner, circumventing the complexity of conventional approaches.

### Grafting-from RNA_21_–CTA via PET-RAFT Polymerization

Given the successful covalent attachment of CTA to RNA, we proceeded with the synthesis of the RNA–polymer conjugate by the “grafting-from” approach via RAFT polymerization (Figure 2A). We selected the PET-RAFT process due to its robustness and great oxygen tolerance, allowing controlled polymerization in very low volumes (50 μL) and at low CTA concentrations (0.05 mM) without prior deoxygenation.^38^ This small-scale controlled polymerization method is particularly advantageous when working with limited quantities of costly synthetic RNA. Employing the synthesized RNA_21_–CTA as a macro-CTA, aqueous PET-RAFT polymerization of oligo­(ethylene oxide) methyl ether methacrylate (OEOMA_500_, average *M*_n_ = 500) was conducted using EY as the organic photocatalyst and triethanolamine (TEOA) as the electron donor. Under green light irradiation, the excited EY reduced covalently attached CTA, generating radicals for direct initiation and chain growth from RNA, yielding the RNA–polymer conjugate. Prior to grafting from RNA–CTA, we optimized the polymerization conditions using CPADB as a model CTA (Tables S5–S7). These model polymerizations demonstrated good MW control and relatively low dispersity even at a low reaction volume (50 μL) and CTA concentration (0.3 mM) without deoxygenation. Under optimized conditions, we then employed RNA_21_–CTA for the synthesis of RNA_21_-*b*-pOEOMA_500_ conjugates. After 1 h of polymerization, monomer conversion reached 26% by ^1^H NMR analysis. Size-exclusion chromatography equipped with a multiangle light scattering detector (SEC-MALS) showed that the trace of the conjugate completely shifted to a higher MW region without tailing or shoulder peaks in the lower MW region (Figure 2B), indicating high initiation efficiency from RNA_21_–CTA. The resulting RNA conjugate had a dispersity of 1.38 and a MW (*M*_n,MALS_) of 790,000, which was in good agreement with the theoretical value (*M*_n,th_ = 846,000). The higher dispersity of the synthesized bioconjugate compared to model reactions using CTA can be attributed to the heterogeneous CTA incorporation per RNA strand (i.e., ranging from 1 to 8 of attached CTA per RNA_21_ by MALDI-TOF and PAGE analysis in Figure 1), consistent with previous observations of grafting-from CTA-modified protein via PET-RAFT polymerization.^38^

**Figure 2:**
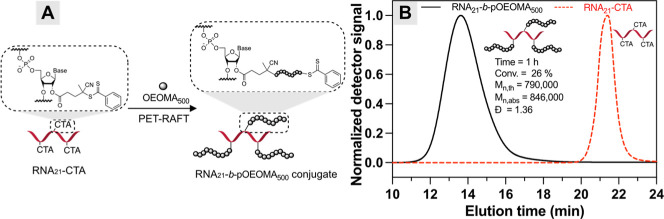
(A) Synthesis of the RNA–polymer conjugate by the “grafting-from” approach via PET-RAFT. (B) SEC-MALS traces of RNA_21_-*b*-pOEOMA_500_ hybrids, compared with RNA_21_–CTA macro-CTA. Reaction conditions: [OEOMA_500_]/[TEOA]/[EY] = 300 mM/0.9 mM/0.015 mM, [RNA_21_–CTA] = 0.05 mM, [CTA] = 0.3 mM.

### “Grafting-from” Biomass RNA via Postsynthetic CTA Modification

Previous approaches for synthesizing NA–polymer conjugates via RAFT polymerization have been limited to synthetic NA, primarily due to the lack of effective and versatile methods for direct CTA incorporation in a postsynthetic manner.^1^ This challenge has hindered the polymer modification of natural RNAs with diverse biological activities (e.g., mRNA, aptamer, and biomass RNA). Expanding RNA substrate scope to these nonsynthetic RNAs would enable the development of novel biomaterials.^4^ Motivated by this, biomass RNA (bmRNA) extracted from *torula* yeast was selected to assess the feasibility of direct CTA modification (Figure 3A). bmRNA was incubated with CTA-AI ([2′-OH]/[CTA-AI] ≈ 30) in a DMSO/H_2_O mixture (20%, v/v) under gentle shaking overnight. In contrast to the gray color of untreated bmRNA, purification of modified bmRNA via precipitation from isopropanol yielded a yellow pellet that could be redispersed in water (Figure 3B), similar to the observation from synthetic RNA modification (Figure S2). Additionally, UV–Vis spectroscopy of bmRNA–CTA also showed a pronounced shoulder peak at 320 nm corresponding to the attached CTA moiety (Figure 3C), suggesting the successful covalent CTA modification of bmRNA.

**Figure 3:**
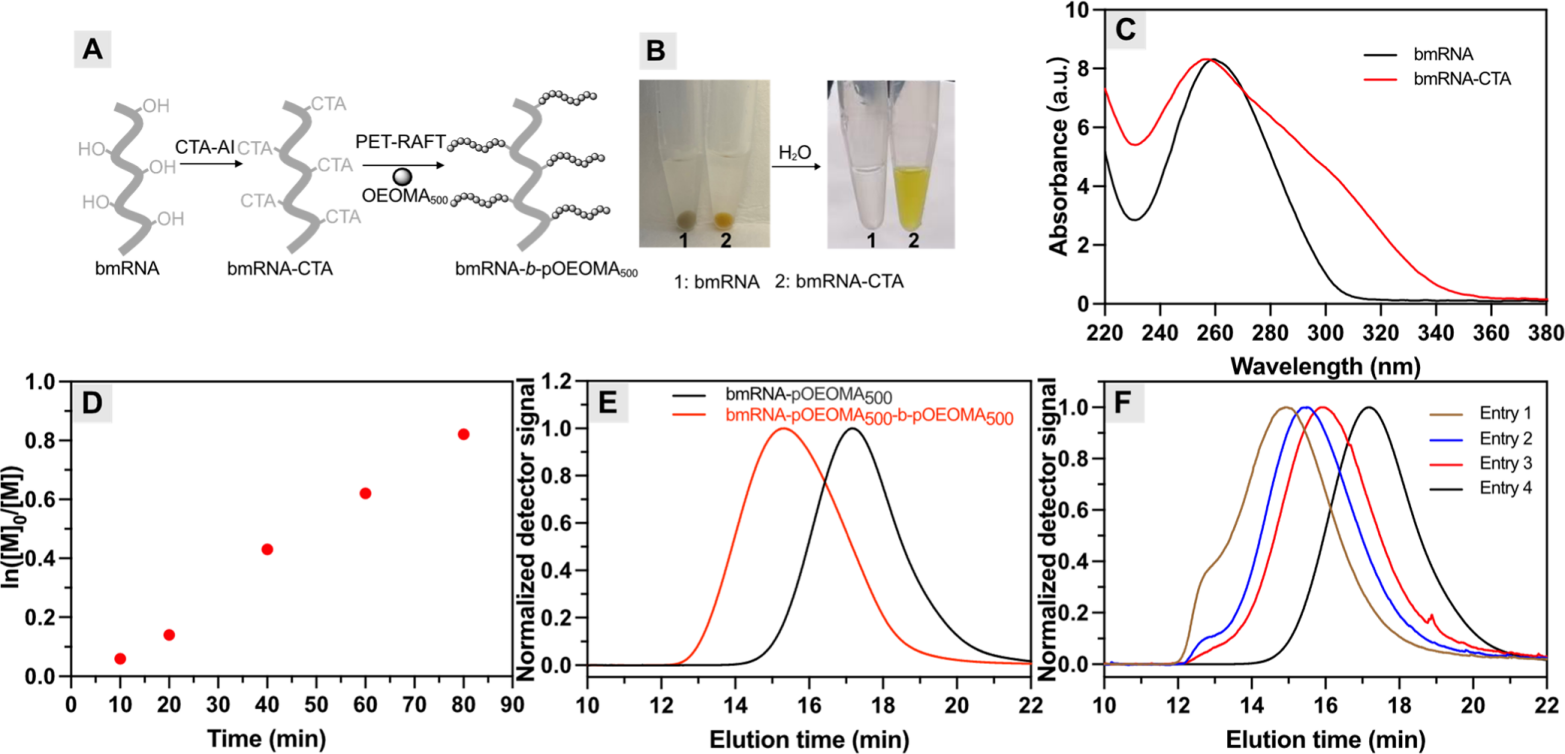
(A) “Grafting-from” approach for synthesizing biomass RNA-*b*-pOEOMA_500_ via PET-RAFT polymerization. (B) Digital images and (C) UV–Vis spectra of bmRNA before and after CTA-AI treatment. (D) First-order kinetic plot. (E) Chain extension of bmRNA-*b*-pOEOMA_500_ with OEOMA_500_. (F) Synthesis of bmRNA-*b*-pOEOMA_500_ with varying molecular weights. Reaction conditions: all polymerizations were conducted under the general polymerization conditions using OEOMA_500_ as the monomer under green light irradiation (Table 1).

The resulting bmRNA–CTA was then tested for mediating the PET-RAFT polymerization of OEOMA_500_ to synthesize biomass RNA conjugates under conditions similar to “grafting-from” RNA_21_–CTA. The kinetic study exhibited a linear semilogarithmic plot after a short induction period (c.a. 5 min, Figure 3D), attributed to the required time for the consumption of oxygen in the reaction mixture. Linear regression of the polymerization kinetic plot yielded the apparent polymerization rate constant at 0.0111 min^–1^, which is consistent with similar PET-RAFT systems under comparable conditions.^39^ To assess the chain-end fidelity of the resulting bioconjugates, bmRNA–pOEOMA_500_ (*M*_n,MALS_ = 310,000, *D̵* = 1.43) was subjected to chain extension with OEOMA_500_. The SEC-MALS analysis showed that the trace of the resulting conjugate clearly shifted to the higher MW region (*M*_n,MALS_ = 1,400,000, *D̵* = 1.57) without a shoulder peak (Figure 3E), confirming chain-end fidelity for reinitiation of RAFT polymerization.

One of the key advantages of the “grafting-from” approach is to control the MW of the resulting conjugates by tuning the targeted degree of polymerization (DP) (DP_t_ = [monomer]/[CTA]). Therefore, polymerizations were conducted by varying the bmRNA–CTA concentration (0.5–6 mg/mL) while maintaining constant concentrations of all other polymerization components. The monomer conversion reached 20–50% within 60 min for all reactions (Table 1, Figure 3F). With increasing concentration of bmRNA–CTA (i.e., lower DP_t_), the *M*_n,MALS_ and DP of the resulting bmRNA–pOEOMA_500_ decreased while maintaining a relatively low dispersity (1.26 ≤ *D̵* ≤ 1.49). These results demonstrate the capability of our approach to engineer the MW of the resulting biomass RNA–polymer conjugates.

**Table 1:** Synthesis of bmRNA-*b*-pOEOMA_500_ with Varying Molecular Weights via PET-RAFT Polymerization^a^

entry	bmRNA–CTA (mg/mL)	conv. (%)^b^	*M*_n,MALS_ ^c^	*D̵* ^c^	DP^d^
1	0.5	50	1,722,000	1.49	3420
2	1.5	31	943,000	1.40	1862
3	3	28	672,000	1.39	1319
4	6	20	347,000	1.26	670

a Reaction conditions: [OEOMA_500_] = 300 mM, [EY] = 0.015 mM, [TEOA] = 0.9 mM, [bmRNA–CTA] = 0.5–6 mg/mL, irradiated under green light LEDs (527 nm, 3.7 mW/cm^2^) for 60 min.

b Monomer conversion was determined by ^1^H NMR spectroscopy.

c Molecular weight (*M*_n,MALS_) and dispersity (*D̵*) were determined by SEC-MALS analysis (phosphate-buffered saline as an eluent).

d Degree of polymerization (DP) for the polymers grafted from bmRNA–CTA was calculated using the following equation: DP = [*M*_n,MALS_ of the conjugate – *M*_n,MALS_ of bmRNA–CTA]/molar mass of OEOMA_500_.

### Functional Biomass RNA–Polymer Nanoassemblies and Hydrogels

The development of renewable polymer materials from biomass sources has gained significant attention in pursuit of more sustainable polymer science. Compared to the widely explored lignin and cellulose counterparts, RNA remains largely unexplored despite its natural abundance.^40^ This underutilization primarily stems from the lack of effective methods for biomass RNA modification.^35^ Leveraging our direct and universal RNA modification via acylation chemistry, we synthesized thermoresponsive bioconjugate nanoassemblies by grafting poly­(*N*-isopropylacrylamide) (pNIPAm) on bmRNA via PET-RAFT polymerization in the presence of bmRNA–CTA (Figure 4A). The resulting bmRNA-*b*-pNIPAm (*M*_n,MALS_ = 209,000, *D̵* = 1.57) was purified through thermal precipitation, followed by dialysis. Transmittance measurements of the purified bioconjugate revealed a lower critical solution temperature (LCST) of 34 °C (Figure 4B). Upon heating above its LCST, dynamic light scattering (DLS) analysis showed a particle size increase from 15 to 252 nm (Figure 4C), attributed to the collapse and aggregation of grafted pNIPAm chains within the bioconjugates. To confirm the synthesis of biomass RNA bioconjugate, we employed SYBR Gold staining, a fluorogenic dye that emits strong fluorescence upon binding to RNA. Fluorescence measurements of the stained conjugates exhibited a significant fluorescence, while negligible fluorescence was observed for pNIPAm alone (Figure 4D), confirming the presence of bmRNA in the conjugates. To rule out the possibility of physical adsorption or encapsulation of bmRNA in precipitated pNIPAm, a physical mixture of bmRNA and pNIPAm in water was subjected to thermal precipitation. The recovered precipitates showed negligible fluorescence after staining, confirming the absence of physical binding of bmRNA and further validating the successful synthesis of covalent conjugates.

**Figure 4:**
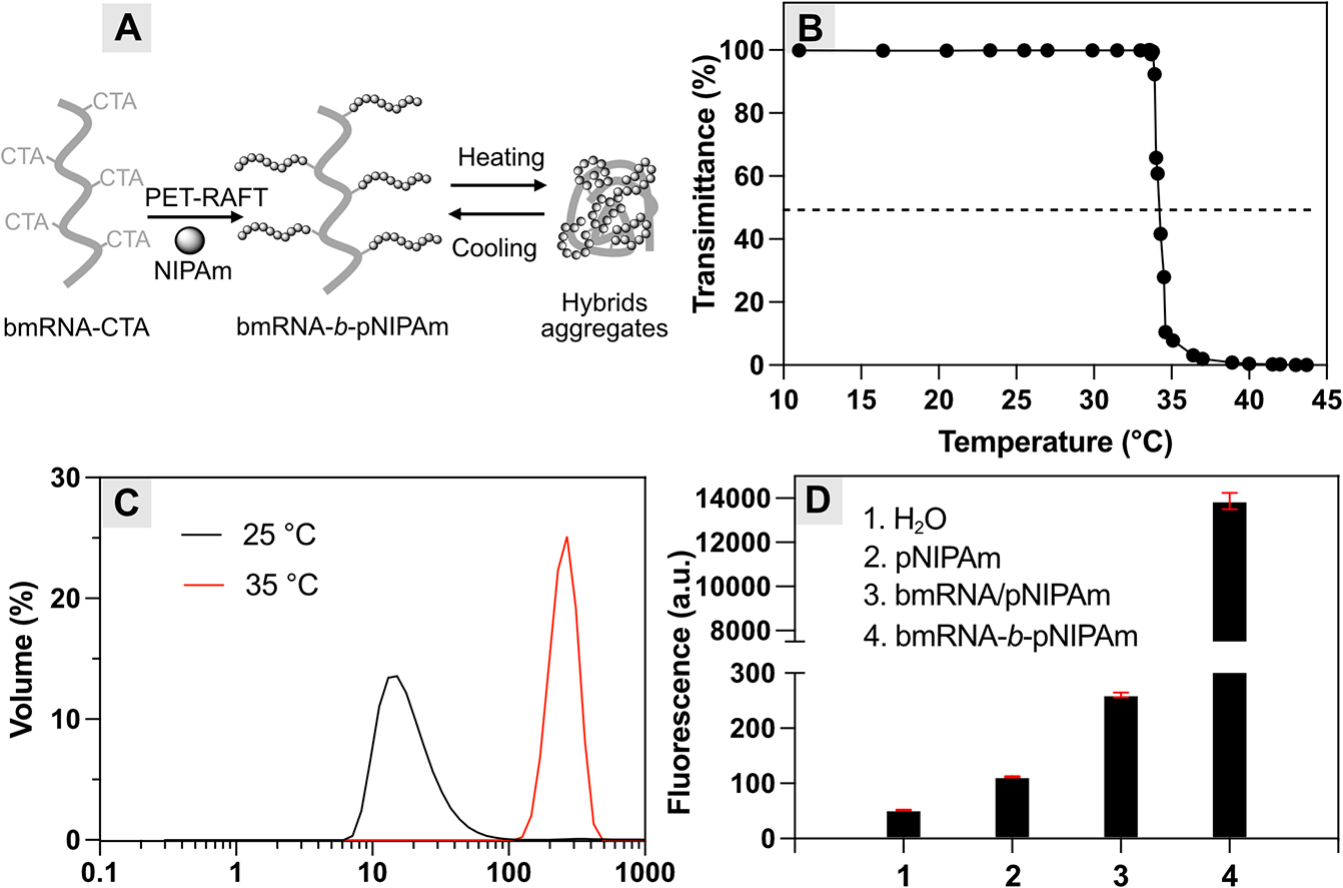
(A) Synthesis of thermoresponsive bmRNA-*b*-pNIPAm nanoassemblies. (B) LCST behavior of bmRNA-*b*-pNIPAm measured by UV–vis at 500 nm. (C) Thermoresponsive size change measured by DLS. (D) Fluorescence intensity of conjugates after staining with SYBR Gold (λ_ex_: 495 nm, λ_em_: 537 nm), compared with control groups H_2_O, pNIPAm, and precipitates from the physical mixture of bmRNA and pNIPAm.

Next, we expanded this approach to fabricate degradable biomass RNA hydrogels via RAFT polymerization in the presence of cross-linkers (Figure 5A). To maximize the RNA content in the hydrogel and enhance its degradability, a bmRNA-derived acrylamide cross-linker was synthesized through acylation chemistry using vinyl acyl imidazole reagents (bmRNA-Am, Figure S6),^35^ which has been successfully applied for constructing RNA hydrogels. PET-RAFT polymerization of OEOMA_500_ in the presence of biomass RNA-derived CTA and cross-linker led to gelation after 120 min of green light irradiation in 96-well plates, forming a cylindrical hydrogel (Figure 5A). Scanning electron microscopy (SEM) imaging of the lyophilized hydrogel revealed a characteristic microporous morphology at the cross-sectional surface (Figure 5B), which is typical of cross-linked hydrogel networks. Additionally, energy-dispersive X-ray (EDX) mapping further demonstrated a uniform distribution of phosphorus (P) and nitrogen (N) elements originating from RNA throughout the hydrogel matrix (Figures 5B and S7), confirming the successful and homogeneous incorporation of bmRNA into the resulting hydrogel. To further validate the presence of bmRNA, the hydrogel was stained by overnight incubation with the RNA-binding dye GelRed (100×, Figure 5C). Following purification by dialysis, the stained hydrogel exhibited strong red fluorescence under the excitation of UV light. Notably, the stained hydrogel retained its structural integrity in H_2_O for 40 days, demonstrating excellent stability. Given the enzymatic degradation capability of nucleic acids, we investigated the biodegradation of the RNA hydrogel by treatment with FBS (15%) in aqueous buffer at 37 °C. The RNA–polymer hydrogel underwent complete degradation within 40 days (Figures 5C and S8). These results highlight the significant potential of biomass RNA–polymer conjugates for the development of sustainable and biodegradable materials for biomedical applications.

**Figure 5:**
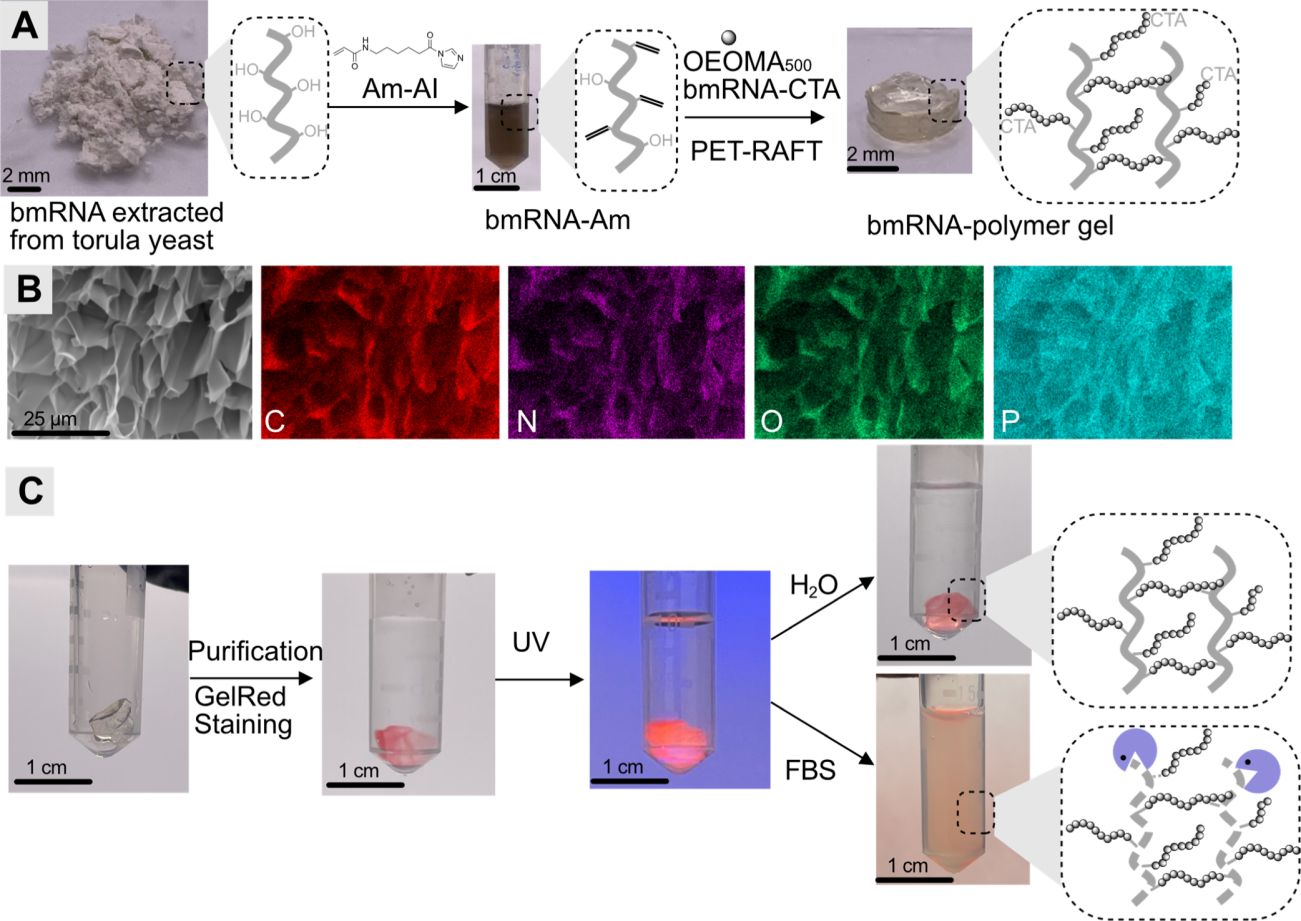
(A) Schematic illustration of fabrication of the RNA–polymer hydrogel via PET-RAFT polymerization in the presence of the synthesized biomass RNA cross-linker (bmRNA-Am) and (B) SEM image and EDX elemental mapping of the lyophilized RNA–polymer hydrogel (scale bar = 25 μm). (C) RNA hydrogel after staining with GelRed (λ_ex_: 320 nm and λ_em_: 537 nm), fluorescence under excitation of UV light (320 nm), and enzymatic degradation of the hydrogel in FBS (15%) compared to that in H_2_O for 40 days.

## Conclusions

In conclusion, we have developed a novel and versatile approach for synthesizing RNA–polymer bioconjugates and functional materials through direct CTA incorporation into RNA, followed by PET-RAFT polymerization. Our strategy employed a CTA-functionalized acyl imidazole reagent (CTA-AI), synthesized by a one-step coupling reaction of commercially available reagents. The nucleophilic reaction of 2′-OH in RNA with CTA-AI facilitated direct and quantitative modifications of various synthetic RNAs with different sequences. Utilizing RNA_21_–CTA as a model macro-CTA, PET-RAFT polymerization generated well-defined RNA–polymer conjugates with controlled MW and low dispersity. Furthermore, this postsynthetic modification approach enabled the first example of successful modification of biomass RNA with polymers via RAFT polymerization, significantly expanding the scope of RNA substrates for bioconjugate synthesis. Additionally, this versatile technique was also applied for fabricating functional bioconjugate materials using widely available biomass RNA, such as thermoresponsive bmRNA-*b*-pNIPAm nanoassemblies and biodegradable hydrogels. This advancement enables direct CTA modification of both synthetic and biomass RNAs through a facile and one-pot reaction using commercially available reagents, overcoming the limitations of tedious and multistep solid-phase synthesis approaches. We envision that this robust approach will significantly broaden access to RNA–polymer conjugates and materials, accelerating their development as advanced biomaterials and biomedicines with combined functionalities of RNAs and synthetic polymers.

## Supplementary Material



## Notes

Additional details of instrumentation and experimental results, including digital camera images of the reaction setup, ^1^H NMR, UV–vis spectra, EDX, and MALDI-TOF results (PDF)
