# Supplementary material for: RNA–Polymer Conjugates via Direct Incorporation of the Chain Transfer Agent and PET–RAFT Polymerization
Source: Biomacromolecules. 2025 Aug 25;26(9):5948–57. doi: 10.1021/acs.biomac.5c00838 (PMC12421687; doi:10.1021/acs.biomac.5c00838)
Supplement: Supplementary file 1 [file bm5c00838_si_001.pdf]

RNA−Polymer Conjugates via Direct Incorporation of the Chain Transfer Agent and PET−RAFT Polymerization

Xiaolei Hu, Jaepil Jeong, Hironobu Murata, Rongguan Yin, Subha R. Das, and Krzysztof Matyjaszewski*

Department of Chemistry, Carnegie Mellon University, Pittsburgh, Pennsylvania 15213, USA

*Correspondence: K.M. (km3b@andrew.cmu.edu)

Table of Contents

Instrumentation 3
RNA synthesis 3
Photoreactor for PET-RAFT 3
Nanodrop UV-Vis spectrophotometer 3
Matrix-assisted laser desorption ionization time-of-flight (MALDI-TOF) mass spectrometry 3
PAGE gel imaging 3
^{1}H Nuclear magnetic resonance (^{1}H NMR) 3
Size exclusion chromatography with a multi-angle light scattering (SEC-MALS) 3
Microplate reader 4
UV-Vis spectrophotometer 4
Dynamic light scattering (DLS) 4
Scanning electron microscope (SEM) 4
Supplementary Data 5
References 13

Instrumentation

RNA synthesis

RNA was synthesized following the previously reported protocol using the MerMade 4 oligonucleotide synthesizer (Bioautomation).[1]

Photoreactor for PET-RAFT

Polymerizations were carried out in a glass insert under green LEDs (520 nm, 3.7 mW/cm^2) except for the hydrogel synthesis in a 96-well plate mounted on a 96-point green light LED array (527 nm, 20 mW/cm^2) with Lumidox Gen II LED Controller. (Analytical Sales and Services, Inc)

Nanodrop UV-Vis spectrophotometer

The absorbance spectrum of RNA is recorded using One UV-Vis spectrophotometer (ThermoFisher Scientific).

Matrix-assisted laser desorption ionization time-of-flight (MALDI-TOF) mass spectrometry

MALDI-TOF spectra of oligonucleotides were recorded by UltrafleXtreme MALDI-TOF Mass Spectrometer (Bruker) using MTP 384 Target Plate Ground Steel (Bruker).

PAGE gel imaging

Polyacrylamide gel imaging was carried out by Typhoon FLA 9000 gel scanner (GE Healthcare Life Sciences).

^{1}H Nuclear magnetic resonance (^{1}H NMR)

^{1}H NMR spectra were recorded on Bruker Avance III 500 MHz spectrometer with D_2O or DMSO-d_6 used as the solvent.

Size exclusion chromatography with a multi-angle light scattering (SEC-MALS)

SEC-MALS measurements of cationic, anionic, and zwitterionic polymers were conducted using the Agilent SEC system (Agilent, 1260 Infinity II with UV detector)

coupled with viscometer, MALS, DLS, and RI detectors (Wyatt Technology, USA). Measurements were performed using the Waters Ultra hydrogel linear column with 1X DPBS as an eluent at a flow rate of 0.5 mL/min and room temperature.

Microplate reader

The fluorescence intensity of dyes was recorded by using the microplate reader (The Infinite® M1000, Tecan).

UV-Vis spectrophotometer

The LCST of bmRNA-pNIPAM was determined by using UV−VIS spectrometer (Lambda 2, PerkinElmer)

Dynamic light scattering (DLS)

Particle sizes of bmRNA-pNIPAM conjugates at different temperatures were determined by using a Zetasizer Nano from Malvern Instruments, Ltd.

Scanning electron microscope (SEM)

SEM and EDX elemental mapping of RNA-polymer hydrogel was performed on a Quanta 600 FEG instrument with XMAX 80mm SDD EDX detector.

Supplementary Data

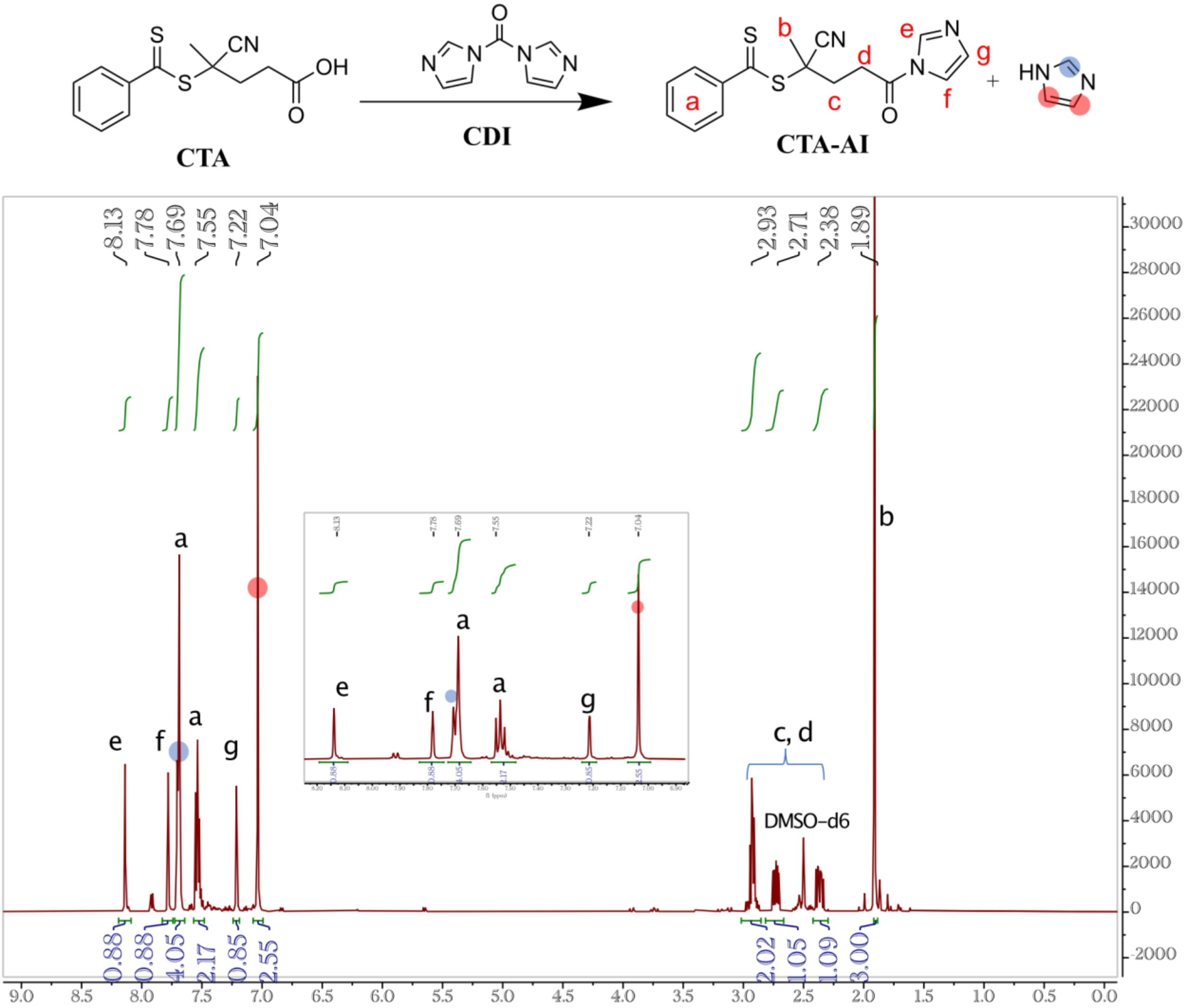

Figure. S1. ^{1}H NMR of crude CTA-AI in DMSO-d$_6$ (500 MHz, DMSO-d$_6$): δ 8.13 (s, 1H), 7.78 (s, 1H), 7.71 (s, 1H), 7.69 (s, 3H), 7.55 (t, *J* = 7.8 Hz, 2H), 7.22 (s, 1H), 7.04 (s, 2H), 2.93 (m, 2H), 2.71 (m, 1H), 2.38 (m, 1H), 1.89 (s, 3H).

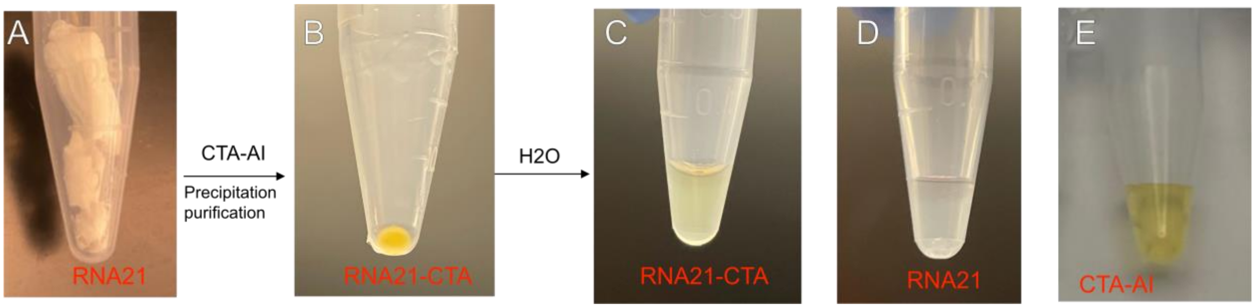

Figure S2. Digital image of RNA_{21} (A) before and (B, C) after treatment of CTA-AI, compared with (D) RNA_{21} solution in H_2O, and (E) CTA-AI solution in DMSO (10 mM).

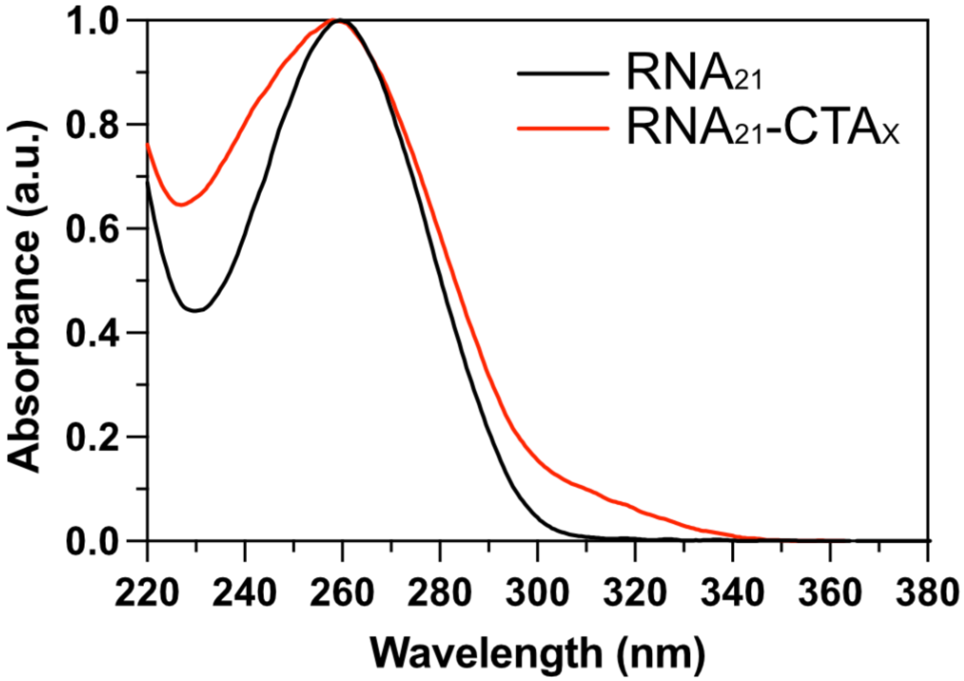

Figure S3. UV-vis spectra of RNA_{21} before and after CTA-AI treatment.

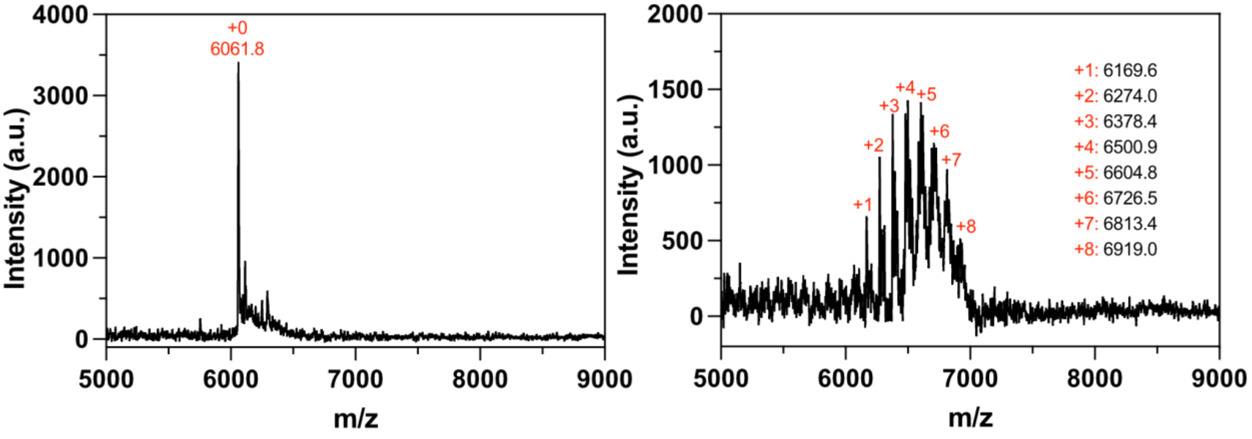

Figure S4. MALDI-TOF spectra of RNA_{20} before and after treatment with CTA-AI (RNA_{20}-CTA); [CTA-AI] / [RNA_{20}] = 650, [CTA-AI] = 130 mM, 20% DMSO in H_2O, incubated at room temperature overnight.

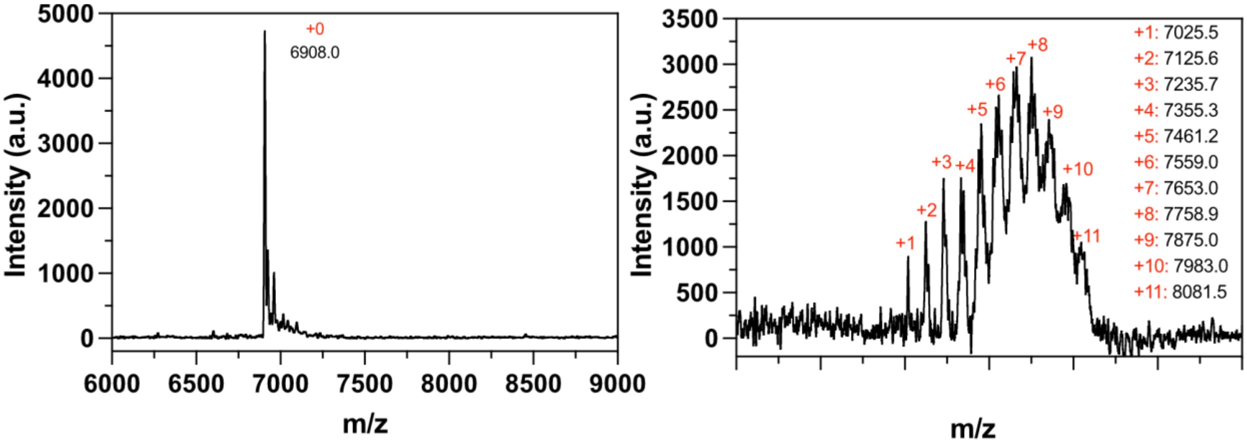

Figure S5. MALDI-TOF spectra of RNA_{22} before (a) and after treatment with CTA-AI (b); [CTA-AI] / [RNA_{22}] = 650, [CTA-AI] = 130 mM, 20% DMSO in H_2O, incubated at room temperature overnight.

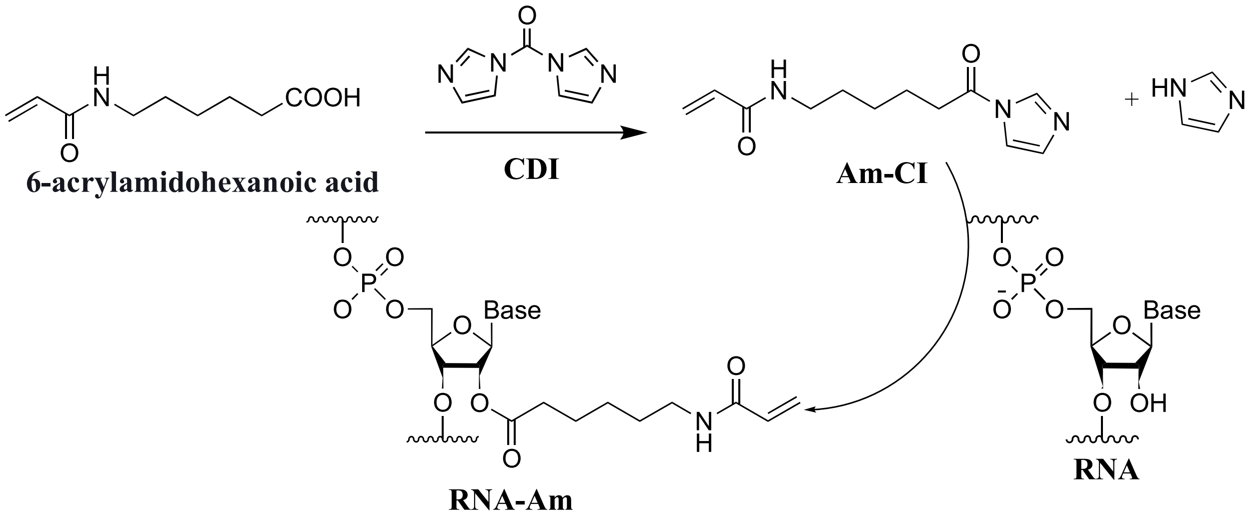

Figure S6. Synthesis of bmRNA-derived acrylamide crosslinker (bmRNA-Am).

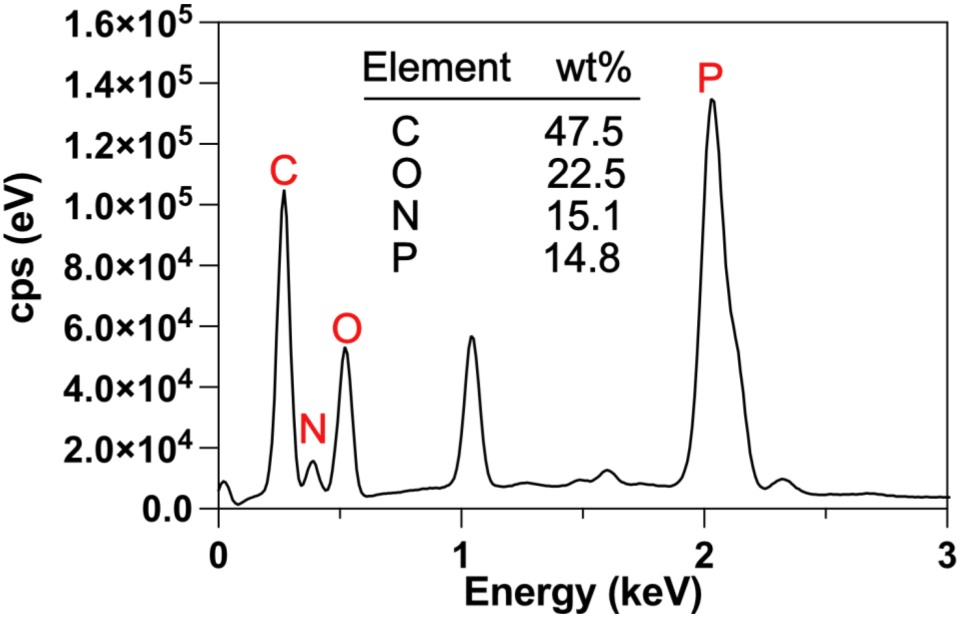

Figure S7. EDX spectra and elemental composition of biomass RNA hydrogels.

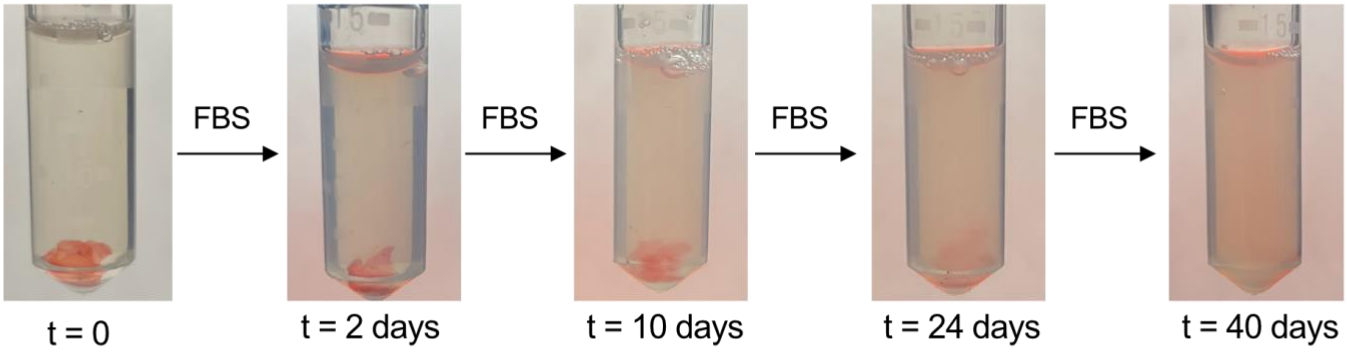

Figure S8. Enzymatic degradation of the hydrogel in FBS (15%) at different times.

Table S1. Sequences of RNA oligonucleotides used in this manuscript.

Name	Sequence (5' to 3')
RNA_{21}	rArCrA rGrCrU rCrUrG rArCrU rGrCrU rCrGrA rCrGrU
DNA_{21}	ACA GCT CTG ACT GCT CGA CGT
RNA_{20}	rUrUrU rUrUrU rUrUrU rUrUrU rUrUrU rUrUrU rUrU
RNA_{22}	rUrArC rUrUrG rUrUrA rGrUrC rUrUrG rUrUrA rGrUrA rC

Table S2. Theoretical MW of RNA_{21}-CTA_x adducts and cleaved product of RNA_{21}-CN_x, compared with $M_{n,MALDI}$ obtained by MALDI-TOF.

Number of incorporated CTA (X)	$M_{n,th}$ of RNA_{21}-CTA_x	$M_{n,th}$ of RNA_{21}-CN_x	$M_{n,MALDI}$
0	6648		6653.4
1	6909	6755	6754.3
2	7170	6862	6860.4
3	7431	6969	6977.6
4	7692	7076	7080.0
5	7953	7183	7187.1
6	8214	7290	7304.6
7	8475	7397	7393.4
8	8736	7504	7512.6

Table S3. Theoretical MW of RNA_{20}-CTA_x adducts and cleaved product $M_{n,th}$ of RNA_{20}-CN_x, and $M_{n,MALDI}$ obtained by MALDI-TOF.

Number of incorporated CTA (X)	$M_{n,th}$ of RNA_{20}-CTA_x	$M_{n,th}$ of RNA_{20}-CN_x	$M_{n,MALDI}$
0	6061		6061.8
1	6322	6168	6169.6
2	6583	6275	6274.0
3	6844	6382	6378.4
4	7105	6489	6500.9
5	7366	6596	6604.8
6	7627	6703	6726.5
7	7888	6810	6813.4
8	8149	6917	6919.0

Table S4. Theoretical MW of RNA_{22}-CTA_x adducts and cleaved product RNA_{22}-CN_x, and $M_{n,MALDI}$ obtained by MALDI-TOF.

Number of incorporated CTA (X)	$M_{n,th}$ of RNA_{22}-CTA_x	$M_{n,th}$ of RNA_{22}-CN_x	$M_{n,MALDI}$
0	6919		6908.0
1	7180	7026	7025.5
2	7441	7133	7125.6
3	7702	7240	7235.7
4	7963	7347	7355.3
5	8224	7454	7461.2
6	8485	7561	7559.0
7	8746	7668	7653.0
8	9007	7775	7758.9
9	9268	7882	7875.0
10	9529	7989	7983.0
11	9790	8096	8081.5

Table S5. Optimization of PET-RAFT polymerization conditions with varying [CTA]

Entry	[CTA] (mM)	Conv.[b] (%)	$M_{n,th}$	[c]$M_{n,app}$	[d]$M_{n,abs}$	[c]Đ
1	1.5	65	64,700	48,200	58,593	1.11
2	0.5	65	195,000	94,800	132,973	1.33
3	0.3	56	280,000	104,000	148,765	1.67

[a]Reactions conditions: [$OEOMA_{500}$]/[TEOA]/[EY] = 300 mM / 0.9 mM / 0.015 mM, [CTA] = 0.5-3 mM, irradiated under green light LEDs for 1h. Monomer conversion was determined by using ^{1}H NMR spectroscopy. [c]Molecular weight ($M_{n,app}$) and dispersity (Đ) were determined by SEC analysis (DMF as eluent) calibrated to polystyrene standards. [d]Absolute molecular weight ($M_{n,abs}$) was determined by Mark-Houwink calibration.

Table S6. Optimization of PET-RAFT polymerization conditions with varying reaction volume at [CTA] = 1.5 mM.[a]

Entry	Volume (μL)	Conv.[b] (%)	$M_{n,th}$	[c]$M_{n,app}$	[d]$M_{n,abs}$	[c]Đ
1	250	65	64,700	48,200	58,593	1.11
2	200	81	81,000	51,200	63,040	1.13
3	150	81	81,000	50,000	61,254	1.12
4	100	70	70,000	45,100	54,058	1.12
5	50	65	65,000	48,100	58,445	1.14

[a]Reactions conditions: [$OEOMA_{500}$]/[TEOA]/[EY] = 300 mM / 0.9 mM / 0.015 mM, [CTA] = 1.5 mM, irradiated under green light LEDs for 1h. Monomer conversion was determined by using ^{1}H NMR spectroscopy. [c]Molecular weight ($M_{n,app}$) and dispersity (Đ) were determined by SEC analysis (DMF as eluent) calibrated to polystyrene standards. [d]Absolute molecular weight ($M_{n,abs}$) was determined by Mark-Houwink calibration.

Table S7. Optimization of PET-RAFT polymerization conditions with varying reaction volume at [CTA] = 0.5 mM.[a]

Entry	Volume (μL)	Conv.[b] (%)	$M_{n,th}$	[c]$M_{n,app}$	[d]$M_{n,abs}$	[c]Đ
1	250	65	195,000	94,800	132,973	1.33
2	200	63	189,000	92,100	128,398	1.30
3	150	63	189,000	86,600	119,168	1.39
4	100	62	186,000	85800	117,836	1.38
5	50	55	165,000	83900	114,681	1.32

[a]Reactions conditions: [$OEOMA_{500}$]/[TEOA]/[EY] = 300 mM / 0.9 mM / 0.015 mM, [CTA] = 0.5 mM, irradiated under green light LEDs for 1h. Monomer conversion was determined by using ^{1}H NMR spectroscopy. [c]Molecular weight ($M_{n,app}$) and dispersity (Đ) were determined by SEC analysis (DMF as eluent) calibrated to polystyrene standards. [d]Absolute molecular weight ($M_{n,abs}$) was determined by Mark-Houwink calibration.

References

1. Jeong, J.; Hu, X.; Murata, H.; Szczepaniak, G.; Rachwalak, M.; Kietrys, A.; Das, S. R.; Matyjaszewski, K., RNA-Polymer Hybrids via Direct and Site-Selective Acylation with the ATRP Initiator and Photoinduced Polymerization. *Journal of the American Chemical Society* **2023,** *145* (26), 14435-14445.
